# Visual word recognition among oldest old people: The effect of age and cognitive load

**DOI:** 10.3389/fnagi.2022.1007048

**Published:** 2022-09-30

**Authors:** Carlos Rojas, Bernardo Riffo, Ernesto Guerra

**Affiliations:** ^1^Department of Health Rehabilitation Sciences, University of Bío-Bío, Chillán, Chile; ^2^Department of Spanish, Universidad de Concepción, Concepción, Chile; ^3^Center for Advanced Research in Education, Institute of Education (IE), Universidad de Chile, Santiago, Chile

**Keywords:** aging, fourth age, word recognition, reaction time, accuracy

## Abstract

During the fourth age, a marked physiological deterioration and critical points of dysfunction are observed, during which cognitive performance exhibits a marked decline in certain skills (fluid intelligence) but good performance of others (crystallized intelligence). Experimental evidence describes important constraints on word production during old age, accompanied by a relative stabilization of speech comprehension. However, cognitive changes associated with advanced aging could also affect comprehension, particularly word recognition. The present study examines how the visual recognition of words is affected during the fourth age when tasks involving different cognitive loads are applied. Through linear regression models, performance was compared between two third-age groups and a fourth-age group on reaction time (RT) and accuracy in naming, priming and lexical decision experiments. The fourth-age group showed a significant RT increase in all experiments. In contrast, accuracy was good when the task involved a low cognitive demand (Experiments 1 and 2); however, when a decisional cognitive factor was included (Experiment 3), the fourth-age group performed significantly worse than the younger third-age group. We argue that the behavior observed among fourth-age individuals is consistent with an unbalanced cognitive configuration, in which the fluid intelligence deficit significantly reduces the speed necessary to recognize words, independent of the cognitive load associated with the test. In contrast, the maintenance in crystallized intelligence improves the accuracy of the process, strengthening linguistic functionality in the advanced stages of old age.

## Introduction

Aging constitutes an increasingly longer period (Baltes, 1998; Höpflinger, 2021), during which a series of functional, structural, and biological changes are observed (Chang et al., 2017; Fletcher et al., 2018). This accelerated aging of world population is a new demographic phenomenon in the history of humanity—there have never been so many people of such an advanced age (Suzman et al., 1995; Sudharsanan and Bloom, 2018). Today, two highly marked stages are distinguished: the third age (60–80 years) and the fourth age (from 80 years onward; Baltes, 1998; Höpflinger, 2017). The latter is a relatively new group, and therefore our knowledge about how such changes (i.e., functional, structural, and biological) take place in these latter stages of aging is limited.

The interplay between cognitive and linguistic skills provides functional support and enhance quality of life even in advanced stages of old age. At the same time, cognitive skills are known to be affected by aging (Henderson and Harris, 2016), impacting processing speed, operational capabilities, and the ability to solve problems logically (the so-called fluid intelligence, see Miller et al., 2010; Mackey et al., 2013; Duncan et al., 2017). Critically, in the fourth age, the physiological decline might be more accentuated with critical points of dysfunction appearing, which in turn might compromise the cognitive and linguistic performance of cognitively healthy older adults more deeply (Margrett et al., 2016). However, aging does not consist solely of a decline. Along with the decrease in fluid intelligence, older adults see a maintenance in their cognitive reserves of experience, knowledge, and vocabulary (i.e., crystallized intelligence), which allow them to compensate for certain deficits (Margrett et al., 2016; Stine-Morrow et al., 2016; Hering et al., 2017). Consequently, cognitive aging presents a functional configuration with aspects that show evident deterioration, while other aspects seem well preserved (DeDe and Knilans, 2016).

In addition, physiological changes and the increase in life expectancy have shaped different developmental stages in old age, making older adults a heterogeneous group (Baltes, 1998). Different studies (Schaie, 2005, 2012; Mitchell et al., 2013; Schubert et al., 2020) have reported that, in general, both third- and fourth-age adults retain their crystallized abilities, while their fluid abilities progressively decline until reaching a minimum level of functioning that leads to a generalized cognitive decline prior to death (Burke and Shafto, 2008; Margrett et al., 2016). Nevertheless, visual and auditory perceptual deficits as well as processing speed deficits are accentuated during the fourth age (Poon et al., 2010; Mitchell et al., 2013; DeDe and Knilans, 2016; Whitson et al., 2018; Mick et al., 2021).

As a result of this decline, understanding and producing language during advanced aging becomes a highly complex task given the multiple levels of processing involved (Abrams and Farrell, 2011; Henderson and Harris, 2016; Davis, 2020). Although the precise limits between the physiological and pathological deterioration of language are still somewhat blurred (Abrams and Davis, 2016; James and Goring, 2018; Davis, 2020), normal aging is known to evidence word production deficits, compromising communication and social interaction (James and Burke, 2000; Margrett et al., 2016; Marini and Andreetta, 2016). Comprehension, in turn, seems to be more stable than production. However, cognitive and sensory changes could affect this level of competence, including word recognition (DeDe and Knilans, 2016; James and Goring, 2018). What remains an open question is what happens with word recognition during the fourth age.

Language comprehension research in third-age older adults have shown that, compared to young adults, older adults are significantly slower yet almost as accurate in recognizing words, both in offline and online experiments, and when word appear in isolation, as well as when they appear in context (Stine-Morrow et al., 2001, 2002; Ratcliff et al., 2004a; DeDe and Knilans, 2016; Hedge et al., 2018). The higher processing costs described in third-age older adults are explained by cognitive slowing associated with the decline in fluid intelligence (Salthouse, 1996, 1999; Schaie, 2005, 2012; Margrett et al., 2016) and sensory impairment (Whitson et al., 2018; Mick et al., 2021). In turn, their maintained response accuracy might be related to their crystallized intelligence (Stern, 2009; Ratcliff et al., 2011; Lojo-Seoane et al., 2014; Margrett et al., 2016; Wulff et al., 2016). Should we predict the same pattern for fourth-age older adults?

Word recognition tasks that involve a decisional cognitive factor (i.e., lexical decision task (LDT): answering “yes” to words or “no” to pseudowords) confirm that older adults are significantly slower than are younger people (Ratcliff et al., 2004a,b; Ratcliff et al., 2011, Hedge et al., 2018). However, recognition is facilitated when the cognitive load is reduced to only one option (i.e., a *go/no-go* lexical decision task, in which the person must respond only “yes” when presented with a word; Gordon, 1983), decreasing reaction time (RT) among all groups (Allen et al., 1991; Perea et al., 2005). According to Balota and Chumbley (1984), the effects of the LDT are modulated by accessory stimuli on the cognitive load of a decisional and post-lexical semantic type, which could increase the complexity of the task and mask the genuine recognition time.

In contrast, tests that involve only the naming of the word to be recognized, such as the naming task, limit the effects of accessory stimuli on cognitive control and reduce the complexity of the test generated by making decisions successively (Balota and Chumbley, 1984; Andrews, 1989, 1992; Lupker et al., 1997; Schilling et al., 1998). The naming task has been shown to be sensitive to lexical variables (e.g., lexical frequency), obtaining similar results as the LDT (Álvarez and Carreiras, 1994; Schilling et al., 1998.). Thus, naming seems an appropriate method, with little additional cognitive load, for evaluating word recognition in an aging population.

On the other hand, both young and old people are susceptible to the influence of *priming* during recognition (Ratcliff et al., 2004a,b; Gold et al., 2009; Cocquyt et al., 2019). Semantic priming, for example, can generate additional lexical effects (delayed semantic activation) that facilitate the recognition of the external signal (Balota et al., 2006) and reduce the amount of sensory analysis required for word recognition (Laver and Burke, 1993), factors that might improve performance in old age (Ratcliff et al., 2004a,b).

Therefore, considering the generalized cognitive decline in the fourth age and the varied cognitive load generated by the different recognition tasks described in the present study we ask: What kinds of visual word recognition processes generate greater difficulties during advanced aging? Is word recognition also affected in a task with minimal accessory stimulus on cognitive load such as naming? To what extent does the involving the presence of decisional cognitive factors associated with LDTs and post-lexical semantics factors present in the priming task impact RT and accuracy during the fourth age?

In general, lexical frequency shows a stable behavior throughout the life cycle and has been widely studied from youth to early stages of aging but not yet in the fourth-age group. Therefore, across experiments, we kept the factor lexical frequency constant obtaining a baseline for the expected performance both in reaction times and accuracy. Thus, we examined whether lexical frequency effects depend on the cognitive demand associated with the applied task.

By contrast, other lexical variables (e.g., positional syllable frequency (PSF), imaginability) have been much less studied across ages (DeDe and Knilans, 2016). In this context, we aimed to obtain data from a number of these variables beyond lexical frequency. Are these difficulties exacerbated if such words are of low imaginability and low PSF. The opposite case should take place when words are highly imaginable and have a high PSF (Álvarez et al., 1998; Farrell and Abrams, 2011; Cuetos et al., 2015). By doing this, we attempted to verify whether the modulation effects of these variables change with aging.

## The present study

In three experiments, we examined how the visual recognition of words is affected during the fourth age in lexical tasks involving different levels of cognitive load. We evaluated the effects of advanced aging on RT and accuracy. Experiment 1 corresponds to a simple naming task with minimal additional cognitive load. Experiment 2 was also a naming task, yet we added two priming conditions, addressing potential post-lexical effects. Experiment 3 used an LDT, which involves the activation of a decisional cognitive factor and categorical post-lexical effects. Each experiment was conducted in an individual session, with a minimum of four-week period separating each session. Across experiments, we evaluated the effects of lexical frequency as a well-established predictor of word recognition, which provides a robust test for the effects of aging on this process. In addition, the effects of PSF (Experiment 1); type of prime (Experiment 2); and imaginability (Experiment 3) were also explored.

We predict that the fluid intelligence deficit will reduce the processing speed during the fourth age (relative to the third age), independent of the cognitive load associated with the task performed. In contrast, unaffected crystallized intelligence should be reflected in a good conceptual performance, showing adequate accuracy in both the early and advanced stages of aging, although its protective effect will decrease in those tasks that involve a greater cognitive load.

## Experiment 1

### Methods

#### Participants

All participants were initially contacted through a link between the university and three local older adults’ clubs. The inclusion criteria were as follows: being 60 years of age or older, having 8 years or more of education, having active aging characteristics (physical, social, and mental well-being), having normal or corrected-to-normal vision and hearing, living in the urban area, and performing the experiments over a period of 8 weeks. The exclusion criteria were as follows: presenting cerebrovascular or neurodegenerative disease, presenting depression or other psychiatric illness, or presenting risk scores in any of the screening tests applied (<21 points on the MoCA (Montreal Cognitive Assessment), >11 points on the Yesavage test, and <4 points in reading comprehension). A large number of older adults were invited to participate (circa 140 people). We aimed for a sample size that could provide at least 2,000 data points per experiment. From those older adults who responded to our invitation, we excluded participants that did not fulfill the inclusion criteria or presented some of the exclusion criteria.

Our final sample consisted in 90 older adults (30 per group). Each group was based on participants’ age: from 60 to 69 years (*M* = 65.73 years, SD = 2.99; *M* = 13.00 years of schooling, SD = 1.23), from 70 to 79 years (*M* = 74.00 years, SD = 2.89; *M* = 13.13 years of schooling, SD = 1.81), and from 80 to 92 (*M* = 82.53 years, SD = 3.10; *M* = 13.03 years of schooling, SD = 1.71). The first two represented the third-age groups and the last, the fourth-age group.

Before participating, all older adults read and signed an informed consent form, approved by the University’s Ethics, Bioethics and Biosafety Committee. The study objectives and benefits were explained to the clubs’ authorities, and subsequently, the older adults willing to collaborate underwent an evaluation to verify correct cognitive and emotional performance using the MoCA (Delgado et al., 2019) and the Yesavage Geriatric Depression Scale (Martínez et al., 2002). In addition, reading comprehension was verified using the Boston comprehension subtest. Finally, selected older adults were invited to the Speech Therapy Laboratory of the University to perform the experimental tasks.

### Materials and design

A 2 × 2 experimental design was implemented, including as factors lexical frequency (high vs. low) and PSF of the first syllable (high-low), orthogonally crossed. The lexical frequency of the words was obtained from the *Spanish Lexical Database*,^1^ and the PSF, through the PSF dictionary by Álvarez et al. (1992). The experiment consisted of 150 trials (see Supplementary Appendix A). It contained 60 two- and three-syllable words (nouns, verbs, and adjectives). Another 60 trials corresponded to ortho-phonologically plausible pseudowords in Spanish of identical length and syllabic structure as the words. Finally, 30 fillers and five practice trials were included.

### Procedure

The experiment took place in an individual room, illuminated and acoustically isolated. Visual materials (words and pseudowords) were presented in the center of a 15.6-inch computer screen using *E-Prime 3.0* software. Participants were instructed to read out loud each of the words and pseudowords as quick as possible and without making mistakes. Each trial began with a star in the center of the screen for 1,000 ms followed by the visual materials, written in capital letters, and randomly presented. Five training trials were given, after which experiment began. Participants’ oral response were recorded, but if no response was given after 10 seconds, the experimenter triggered the next trial. Using the *Chronos* voice key, the *E-Prime* software controlled the time elapsing from the presentation of the stimulus until the participant responded orally obtaining trials’ reaction times. The experiment was administered in two blocks, divided by a short break. The whole experiment took 20–25 minutes, approximately.

### Data analysis

We counted the number of correct and incorrect trials bases on participants’ recordings. Trials where the response was the product of involuntarily activating the voice key were considered invalid (2.28% of the experiment). For the RT of each trial, we used a criterion similar to Ratcliff et al., 2004a,b, 2011 where times outside of an interval between 200 and 6,000 ms were excluded. Prior to the inferential analysis, the RT data were log-transformed to approach normality. The statistical analysis was performed using regression models with mixed cross effects, implemented in R (R Core Team, 2020) with the *lme4* (Bates et al., 2015) and *lmerTest* (Kuznetsova et al., 2017) R packages. Such models allowed the inclusion of the intrinsic variability at the participant and item levels (Clark, 1973) in a single regression without the need to aggregate the data. For the RT data, we fitted linear mixed-effect regression models, and for the accuracy data we used generalized linear mixed-effect regression models. The models evaluate the effects of three factors on RT and accuracy: age group (60–69, 70–79, and 80 years or more), lexical frequency (high-low), and PSF (high-low).

All models included the interactions between the fixed effects, random intercepts for both participants and items, and random slopes justified by the design. Since the focus of the present research is the fourth-age group, we used a treatment contrast with this group as the regression intercept, thus evaluating the effect of each predictor on this group primarily and comparing this group directly with the third-age groups. For the predictors with two levels (i.e., lexical frequency and PSF), we used a sum contrast instead.

### Results

**FIGURE 1:**
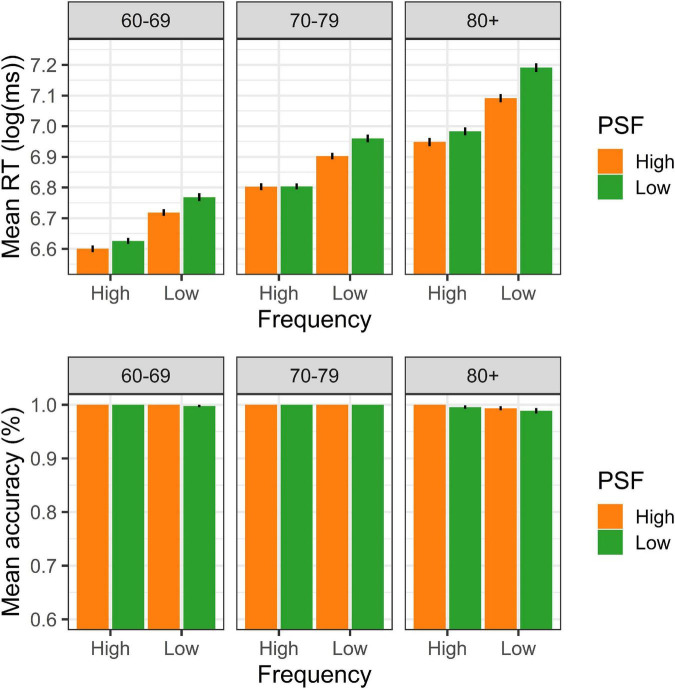
Word response pattern on RT (log) and accuracy in Experiment 1.

The mixed linear regression for the words (Table 1) showed a main effect of the age factor on the RT. Specifically, those in the fourth-age group obtained significantly higher RT (slower responses) than did the groups aged 60–69 (β = –0.178, SE = 0.022, *t* = –8.238, *p* < 0.00) and 70–79 years (β = –0.089, SE = 0.021, *t* = –4.125, *p* < 0.00). In addition, the fourth-age group exhibited main effects on lexical frequency and PSF, which reflected facilitation for words of high lexical frequency over low-frequency words and words of high- compared to low-PSF (Frequency: β = –0.051, SE = 0.007, *t* = –6.879, *p* < 0.00; PSF: β = –0.016, SE = 0.007, *t* = –2.210, *p* = 0.031). Moreover, we observed interaction effects with both third-age groups and lexical frequency (60–69: β = 0.007, SE = 0.003, *t* = 2.176, *p* = 0.033; 70–79: β = 0.008, SE = 0.003, *t* = 2.472, *p* = 0.015), which confirms that, at older ages, the RT difference was greater between the high- and low-frequency words (see Figure 1). Finally, an interaction was observed in the third-age group aged 70–79 years with the PSF (β = 0.006, SE = 0.002, *t* = 2.568, *p* = 0.012). This interaction effect reflects that among the high-lexical frequency words, words with high-PSF words were significantly faster than those of low-PSF in the fourth-age group, while there was no difference between them in the 70–79 years group.

**TABLE 1:** Linear mixed-effects regression reaction time results for Experiment 1.

	Estimate	SE	*t*	Pr*(*>*| t|)*	
Intercept (Group 80+)	6.866	0.021	326.549	0.000	***
Group 60–69	–0.178	0.022	–8.238	0.000	***
Group 70–79	–0.089	0.021	–4.125	0.000	***
Frequency	–0.051	0.007	–6.879	0.000	***
PSF	–0.016	0.007	–2.210	0.031	*
Group 60–69: Frequency	0.007	0.003	2.176	0.033	*
Group 70–79: Frequency	0.008	0.003	2.472	0.015	*
Group 60–69: PSF	0.005	0.003	1.811	0.076	
Group 70–79: PSF	0.006	0.002	2.568	0.012	*
Frequency: PSF	0.006	0.005	1.221	0.227	
Group 60–69: Frequency: PSF	–0.002	0.002	–1.268	0.210	
Group 70–79: Frequency: PSF	0.000	0.002	–0.285	0.776	

***p < 0.001; **p < 0.01; *p < 0.05.

For the accuracy data (Table 2), the generalized mixed effects regression evidenced no significant differences between groups or based on lexical attributes. This could be associated with the low variability generated by the high level of accuracy of the responses (ceiling effect) in all groups (over 98%).

**TABLE 2:** Generalized linear mixed-effects regression accuracy results for Experiment 1.

	Estimate	SE	*z*	Pr*(*>*| z|)*
Intercept (Group 80**+**)	25.888	30.421	0.851	0.395
Group 60–69	4.860	22.828	0.213	0.831
Group 70–79	3.673	19.996	0.184	0.854
Frequency	1.267	22.077	0.057	0.954
PSF	1.320	19.288	0.068	0.945
Group 60–69: Frequency	–0.494	23.913	–0.021	0.984
Group 70–79: Frequency	–1.747	21.200	–0.082	0.934
Group 60–69: PSF	–0.355	24.542	–0.014	0.988
Group 70–79: PSF	–1.754	21.952	–0.080	0.936
Frequency: PSF	0.109	21.467	0.005	0.996
Group 60–69: Frequency: PSF	–1.860	17.788	–0.105	0.917
Group 70–79: Frequency: PSF	–0.460	21.777	–0.021	0.983

***p < 0.001; **p < 0.01; *p < 0.05.

### Discussion

In Experiment 1, we assessed word recognition in a sample of older adults using a simple naming task with minimal additional cognitive load. The results showed that the difficulties experienced during the fourth age responded exclusively to an increase in the RT necessary to access the lexical representation, and not in the accuracy of word recognition. In this regard, old adults in the fourth age exhibited a behavior consistent with the changes and cognitive counterbalance described with regards to aging (Margrett et al., 2016). Previous experiments have already reported similar effects in the early stages of old age (Allen et al., 1991; Ratcliff et al., 2004a,b, 2011; Hedge et al., 2018). However, these studies compared young people with older adults—where cognitive differences are strongly marked—without focusing on fourth-age individuals, and using tasks of greater cognitive load (such as LDT).

Specifically, the group main effects observed in Experiment 1 showed that when the task activated only lexical mechanisms and had a low overall cognitive load, the older, fourth-age individuals were slower in recognizing words compared to younger third-age peers (both groups). The generalized cognitive slowing (Salthouse, 1996, 1999) and perceptual deficits that affect vision and hearing (DeDe and Knilans, 2016; Whitson et al., 2018; Mick et al., 2021), which could be related to slower responses, might continue to increase even late in life. Thus, these factors could be underlying, broadly speaking, the systematic RT increase when recognizing a verbal stimulus during the fourth age.

By contrast, the fourth-age group evidenced a high level of accuracy (over 98% in all groups), not different from that observed in the other age groups. In healthy cognitive conditions, older adults can present good conceptual performance, allowing them to recognize and name words with a high rate of accuracy (Ratcliff et al., 2011; DeDe and Knilans, 2016; Wulff et al., 2016). This good performance is associated with the maintenance of semantic capacities during aging (Stern, 2009; DeDe and Knilans, 2016; Wulff et al., 2016), skills that seem to operate autonomously at the speed with which the process is executed. Therefore, the cognitive changes typical of aging, responsible for the marked contrast between the decline in certain skills and the maintenance of others. These effects were evident in the naming task that involved the activation of mainly lexical mechanisms.

Additionally, all older adults exhibit greater difficulty when recognizing low-frequency words. In general, words of low lexical frequency are recognized more slowly because they are not easily accessible in the semantic memory since they have fewer interconnections between their sublexical units and lower activation potential (Perea et al., 2005; Igoa, 2009; Cuetos et al., 2015). In this sense, the interactions between lexical frequency and age groups reflect that for the fourth-age participants, the frequency effect is even more pronounced (i.e., larger processing time difference between high and low frequency words) compared to the third-age groups. Regarding the PSF, older adults from each group accessed words with high PSFs faster relative to low PSF. However, as for the lexical frequency effect, this difference is more pronounced in the oldest older adults (>80 years of age) compared to the other groups. Word whose initial syllable is of a high frequency shares this syllable with many other words (Farrell and Abrams, 2011), so its transmission routes would be more stable in the face of the deficits typical of aging facilitates, in turn, its availability, activation, and recognition.

In synthesis, the results of Experiment 1 suggest that the modulation effects of lexical frequency and PSF described in the literature appear to be maintained in advanced aging, at least, when the recognition task mainly involves the activation of lexical mechanisms. The purpose of Experiment 2 was to stress this hypothesis by adding an additional post lexical load to the process. With that aim, we implemented a priming experiment consisting again of a naming task, with the difference that this time words were preceded by other words (primes) that facilitates or interferes in the target processing (Meyer and Schvaneveldt, 1971). Specifically, we assessed the influence of the semantic and ortho-phonological relations between the prime and the target word on target word recognition. Assuming that the underlying mechanism to name the target word is determined by the recognition of the lexical representations that correspond to the sensory input, this process is free of a decisional cognitive factor. However, it adds a post lexical effect since the activation of a related (i.e., semantic, or ortho-phonological) lexical representation by the prime might exert its influence only after word recognition.

## Experiment 2

### Methods

#### Participants

The sample of participants from Experiment 1 also completed the Experiment 2.

### Materials and design

Experiment 2 was a 2 × 3 design that combined the lexical frequency (high-low) of the target word (that was read out loud) and the type of prime that preceded it (semantic, ortho-phonological, or neutral). As for Experiment 1, the lexical frequency was controlled through the *Spanish Lexical Database*. Prime-target relations were established through a normative study with 20 older adults (different from the participants) who evaluated (on a scale of 1–7) the level of semantic association or ortho-phonological pairs that had previously configured target and prime word pairs (1: “no semantic relationship/no ortho-phonological similarity”; 7: “strong semantic relationship/strong ortho-phonological similarity”). After the norming study, 80 target-prime pairs that obtained the best association scores were selected for the final set. We produced two experimental lists consisting of 100 trials (see Supplementary Appendix B); in one list 40 target words of high lexical frequency where matched with 10 semantic primes, 10 ortho-phonological primes and 20 neutral primes, while the other 40 words had low lexical frequency and were also matched with 10 semantic primes, 10 ortho-phonological primes and 20 neutral primes. The other list rotated the neutral primes with the semantic and ortho-phonological primes. Finally, the experiment included 20 filler words and 10 practice trials were also included at the beginning of each list.

### Procedure and data analysis

The procedure for Experiment 2, was identical to that implemented for Experiment 1, except for the presentation of primes for a 1,000 ms before the onset of the target words. Participants were instructed to silently read the primes and to read the target words out loud. Data analysis was also identical to that in Experiment 1 (except for the predictors included in the models); we removed invalid trials (3.44%), and RT were log-transformed. Both log-RT and accuracy data were analyzed using hierarchical regression models that include age group, lexical frequency, and prime type (i.e., semantic, ortho-phonological, neutral) as fixed effects. The models also incorporated the interactions between the fix effects, random intercepts at the participant and item levels, and random effect structure justified by the design. Using a treatment contrast scheme, the fourth-age group and the neutral prime condition were used as an intercept, comparing this group directly with other participants in their age groups and the various predictive factors.

### Results

Table 3 presents the results from the regression model on participants’ RT. We observed a significant effect of age on this dependent variable, where the two third-age groups had faster responses (lower RTs) than those in the fourth-age group (Group 60–69: β = –0.291, SE = 0.038, *t* = –7.754, *p* < 0.00; Group 70–79: β = –0.151, SE = 0.038, *t* = –4.010, *p* < 0.00). In addition, the fourth-age group exhibited a main effect on the lexical frequency (β = –0.071, SE = 0.010, *t* = –6.944, *p* < 0.00); high-frequency words were read faster than low-frequency words.

**TABLE 3:** Linear mixed-effects regression reaction time results for Experiment 2.

	Estimate	SE	*t*	Pr*(*>*|t|)*	
Intercept (Fourth age)	6.971	0.027	254.873	0.000	***
Group 60–69	–0.291	0.038	–7.754	0.000	***
Group 70–79	–0.151	0.038	–4.010	0.000	***
Frequency	–0.071	0.010	–6.944	0.000	***
Semantic prime	–0.051	0.012	–4.305	0.000	***
Ortho-phonological prime	0.045	0.013	3.471	0.001	***
Group 60–69: Frequency	0.030	0.011	2.749	0.007	**
Group 70–79: Frequency	0.009	0.011	0.821	0.413	
Group 60–69: Semantic prime	0.025	0.016	1.562	0.121	
Group 60–69: Ortho-phonological prime	–0.015	0.018	–0.835	0.406	
Group 70–79: Semantic prime	0.028	0.016	1.714	0.090	
Group 70–79: Ortho-phonological prime	0.000	0.018	0.012	0.990	
Frequency: Semantic prime	0.019	0.011	1.668	0.095	
Frequency: Ortho-phonological prime	0.003	0.012	0.291	0.771	
Group 60–69: Frequency: Semantic prime	–0.016	0.015	–1.057	0.291	
Group 60–69: Frequency: Ortho-phonological prime	0.001	0.016	0.096	0.924	
Group 70–79: Frequency: Semantic prime	0.012	0.016	0.757	0.449	
Group 70–79: Frequency: Ortho-phonological prime	–0.011	0.016	–0.697	0.486	

***p < 0.001; **p < 0.01; *p < 0.05.

**FIGURE 2:**
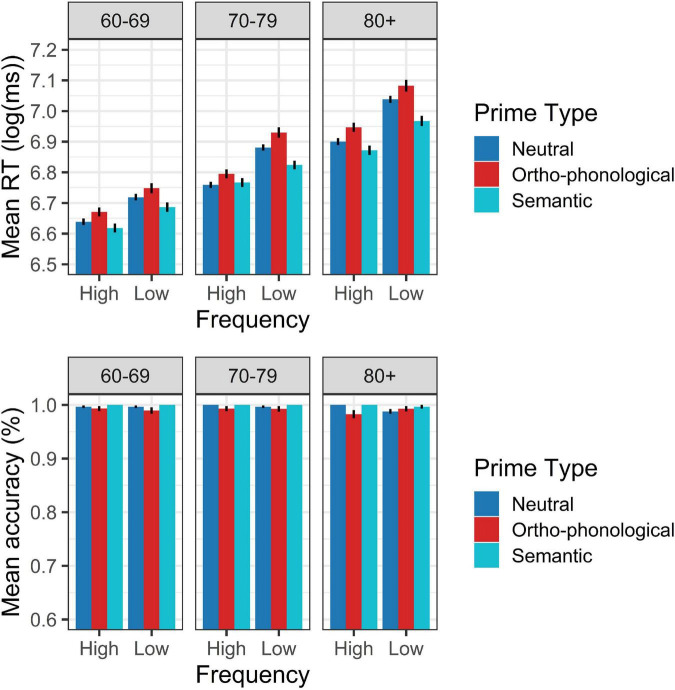
Word response pattern on RT (log) and accuracy in Experiment 2.

The fourth-age group also evidenced effects of both prime types; the semantic prime facilitated lexical access compared to neutral primes (β = –0.051, SE = 0.012, *t* = –4.305, *p* < 0.00), while the ortho-phonological prime exerted an interference effect compared to neutral primes (β = 0.045, SE = 0.013, *t* = 3.471, *p* = 0.001). The regression model also showed an interaction effect between the 60–69-year-old group and lexical frequency (β = 0.030, SE = 0.011, *t* = 2.749, *p* < 0.007), which reflects that, independent of the prime type, a larger difference between frequent and infrequent words was observed in the fourth-age group. Regarding the accuracy of the answers, the generalized linear regression of Table 4 does not show significant differences between accuracy by age group or between the different lexical variables evaluated due to the low variability of the data that generated the high accuracy of the answers obtained in all groups (over 98%, Figure 2).

**TABLE 4:** Generalized linear mixed-effects regression accuracy results for Experiment 2.

	Estimate	SE	*z*	Pr*(*>*|t|)*
Intercept (fourth age)	16.881	38.967	0.433	0.665
Group 60–69	–8.482	38.961	–0.218	0.828
Group 70–79	0.665	78.930	0.008	0.993
Frequency	9.654	38.953	0.248	0.804
Semantic prime	3.378	117.220	0.029	0.977
Ortho-phonological prime	–9.445	38.957	–0.242	0.808
Group 60–69: Frequency	–9.792	38.957	–0.251	0.802
Group 70–79: Frequency	–0.421	78.930	–0.005	0.996
Group 60–69: Semantic prime	17.914	381.559	0.047	0.963
Group 60–69: Ortho-phonological prime	8.524	38.963	0.219	0.827
Group 70–79: Semantic prime	8.661	137.602	0.063	0.950
Group 70–79: Ortho-phonological prime	–0.607	78.930	–0.008	0.994
Frequency: Semantic prime	–3.469	117.188	–0.030	0.976
Frequency: Ortho-phonological prime	–10.319	38.954	–0.265	0.791
Group 60–69: Frequency: Semantic prime	0.645	557.927	0.001	0.999
Group 60–69: Frequency: Ortho-phonological prime	10.588	38.958	0.272	0.786
Group 70–79: Frequency: Semantic prime	–8.593	141.845	–0.061	0.952
Group 70–79: Frequency: Ortho-phonological prime	0.974	78.929	0.012	0.990

***p < 0.001; **p < 0.01; *p < 0.05.

### Discussion

The results of Experiment 2 confirm our findings from Experiment 1. All older adults were faster when they recognized the high-frequency words. Moreover, the deficit in the visual recognition of words for individuals in the fourth age responds to a systematic increase in the time necessary to recognize the stimulus, yet, with adequate processing accuracy. Although no decision was required in either test, Experiment 2 added greater post lexical demand compared with Experiment 1 due to the primes. Notwithstanding, the responses from participants in the fourth-age group were symmetrical to Experiment 1, exhibiting a marked difference between RT (delayed) and accuracy (maintained), consistent with the cognitive changes described in the advanced stages of old age.

Independent of the systematic increase in RT in our experimental group, word recognition was facilitated when the target was preceded by a semantic prime. However, word recognition was inhibited when the prime was ortho-phonological. The effect of the semantic prime is consistent with that reported by Gold et al. (2009), Laver and Burke (1993), Myerson et al. (1992) and Ratcliff et al. (2004a). These authors stated that this behavior could remain stable during aging. Experiments with phonological and/or orthographic prime in young populations describe an RT increase when the prime and target share the initial syllable, interfering with selecting the target, given the morphological similarity between the two (Dufour and Peereman, 2003, 2004). In addition, if the prime and target are two-syllable words, the initial segment of the prime activates the lexical representation of the target. However, this activation does not last because the rest of the phonological information of the prime causes its deactivation, generating an inhibition effect (Spinelli et al., 2001).

Experiments 1 and 2 show that significant differences between those in the third- and fourth-age groups in terms of RT and no differences in terms of accuracy. However, these results were observed in tasks that do not incorporate a conscious cognitive factor that add greater complexity to the process. To further verify these effects, we implemented a task that adds a decisional component. In the LDT, participants must quickly decide whether the stimulus presented is a word from their language. To perform the task, participants must consult in the visual lexical module (Patterson and Shewell, 1987) whether the sequence of letters processed corresponds to some lexical representation stored in their mental lexicon. According to Balota and Chumbley (1984), when young adults perform a LDT, they reach RTs between 700 and 1,500 ms, more than double the RT in normal reading (250 ms). These data suggest that the RT obtained in the LDT not only includes lexical access but also incorporates the time spent in making a decision. For Balota and Chumbley (1984), the cognitive task of making a decision occurs after recognition; therefore, it would influence not only the total RT but also the total cognitive load of the task, making it more complex than other recognition tests. In summary, a basic assumption of the LDT is that the time necessary for the participants to make a decision and respond is determined by recognizing the representations that correspond to the sensory input. This time will be influenced by the accessory cognitive load of the test, which may (or may not) further increase the RT and reduce accuracy among those in the fourth-age group.

## Experiment 3

### Methods

#### Participants

The sample of participants from Experiment 1 and 2 completed the Experiment 3.

### Materials and design

We implemented a 2 × 2 design that combined two lexical frequency levels (high-low) and two imaginability levels (high-low). The lexical frequency of the words was controlled using the *Spanish Lexical Database.* The imaginability level was controlled through a normative study with 20 older adults (different from the participants) who had to evaluate (on a scale of 1–7) how imaginable the word presented was (1: “very difficult to imagine”; 7: “very easy to imagine”). The 120 words that obtained the highest indices of high and low imaginability were selected for the final set. The experiment presented 150 trials (see Supplementary Appendix C). It contained 60 words (nouns, verbs, and adjectives), 30 of high and 30 of low lexical frequency, subdivided into 15 of high and 15 of low imaginability. Another set of 60 trials corresponded to ortho-phonologically plausible pseudowords for Spanish of identical length, conformation, and syllabic structure as the words. Finally, 20 filler trials (randomized together with the experimental lists), while 10 practice trials were also included at the beginning of each list.

### Procedure and data analysis

The procedure and data analysis were the almost same as in Experiment 1. Unlike Experiment 1, participants were instructed to decide whether the stimulus presented was a word or not through the oral response “yes” for words and “no” for pseudowords, rather than reading the words out loud. We removed invalid responses (4.42% of total experimental data), and RT data were transformed with logarithmic function. As in the first two previous experiments, we analyzed our data using generalized and linear mixed models implemented in R (R Core Team, 2020). Regression models included three fixed effects: age group (60–69/70–79/80–92 years), lexical frequency (high-low) and imaginability (high-low). All models incorporated the interactions between the fixed effects and included random intercepts at the participant and item levels and random slopes justified by the design. The fourth-age group was used as an intercept, comparing this group directly with the third-age groups and the various predictive factors.

### Results

**FIGURE 3:**
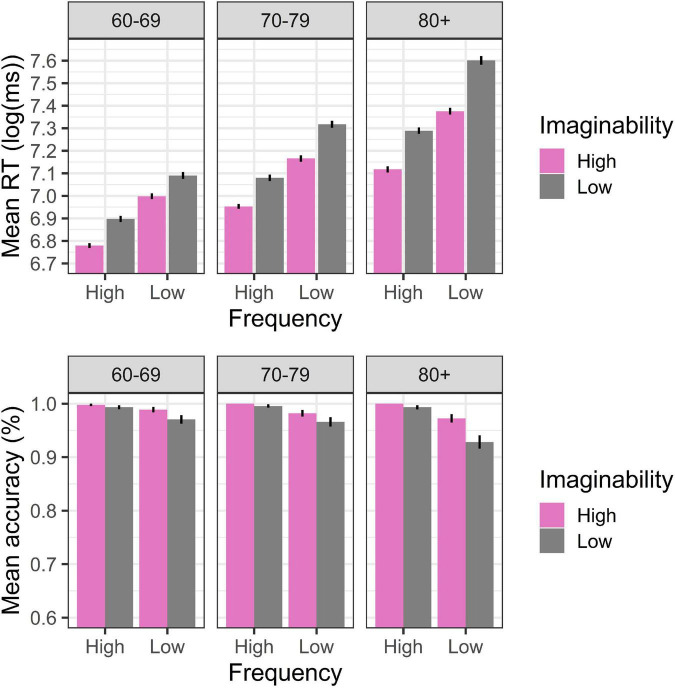
Word response pattern on RT (log) and accuracy in Experiment 3.

The mixed linear regression on participants’ RT (Table 5) shows that the fourth-age group was significantly slower compared to both third-age groups (Group 60–69: β = –0.197, SE = 0.020, *t* = 87.496, *p* < 0.00; Group 70–79: β = –0.108, SE = 0.020, *t* = 86.637, *p* < 0.00). In addition, the fourth-age group exhibited significant effects of lexical frequency and imaginability, obtaining faster RTs for high vs. low-frequency words and for concrete rather vs. abstract words (Frequency: β = –0.087, SE = 0.009, *t* = 71.950, *p* < 0.00; Imaginability: β = –0.054, SE = 0.009, *t* = 65.311, *p* < 0.00). Two-way interaction effects were observed between both third-age groups and lexical frequency and imaginability (Group 60–69: Frequency: β = 0.015, SE = 0.005, *t* = 78.596, *p* = 0.003; Group 70–79: Frequency: β = 0.012, SE = 0.005, *t* = 75.212, *p* = 0.016; Group 60–69: Imaginability: β = 0.017, SE = 0.004, *t* = 66.508, *p* < 0.00; Group 70–79: Imaginability: β = 0.011, SE = 0.004, *t* = 63.238, *p* = 0.009), reflecting that at an older age, the RT difference is greater between the high- and low-frequency words and the concrete compared to the abstract words (Figure 3). Finally, a three-way interaction was also observed between lexical frequency, imaginability and the 60–69 age group (β = –0.005, SE = 0.002, *t* = 48.469, *p* = 0.028). This effect is mostly driven by the large difference between RTs to low frequency low imaginability words and the other experimental conditions, asymmetry that is absent in the 60–69 age group (Figure 3).

**TABLE 5:** Linear mixed-effects regression reaction time results for Experiment 3.

	Estimate	SE	*t*	Pr*(*>*| t|)*	
Intercept (fourth age)	7.142	0.021	132.637	0.000	***
Group 60–69	–0.197	0.020	87.496	0.000	***
Group 70–79	–0.108	0.020	86.637	0.000	***
Frequency	–0.087	0.009	71.950	0.000	***
Imaginability	–0.054	0.009	65.311	0.000	***
Group 60–69: Frequency	0.015	0.005	78.596	0.003	**
Group 70–79: Frequency	0.012	0.005	75.212	0.016	*
Group 60–69: Imaginability	0.017	0.004	66.508	0.000	***
Group 70–79: Imaginability	0.011	0.004	63.238	0.009	**
Frequency: Imaginability	0.003	0.006	57.821	0.658	
Group 60–69: Frequency: Imaginability	–0.005	0.002	48.469	0.028	*
Group 70–79: Frequency: Imaginability	–0.002	0.002	45.487	0.291	

***p < 0.001; **p < 0.01; *p < 0.05.

On the other hand, the generalized linear regression of Table 6 showed significant differences between the accuracy of the fourth-age group and that of the 60–69 age group (β = 0.365, SE = 0.176, *z* = 2.073, *p* = 0.038). We also observed a main effect of the frequency (β = 0.774, SE = 0.232, *z* = 3.3391, *p* = 0.001) and imaginability (β = 0.570, SE = 0.248, *z* = 2302, *p* = 0.021) variables in the fourth-age group, which showed that errors increased significantly in the presence of the low-frequency and abstract words (Frequency: β = 0.774, SE = 0.232, *z* = 3.339, *p* = 0.001; Imaginability: β = 0.570, SE = 0.248, *z* = 2.302, *p* = 0.021).

**TABLE 6:** Generalized linear mixed-effects regression accuracy results for Experiment 3.

	Estimate	SE	*z*	Pr*(*>*|z|)*	
Intercept (Fourth age)	4.673	0.243	19.249	0.000	***
Group 60–69	0.365	0.176	2.073	0.038	*
Group 70–79	0.189	0.169	1.120	0.263	
Frequency	0.774	0.232	3.339	0.001	***
Imaginability	0.570	0.248	2.302	0.021	*
Group 60–69: Frequency	–0.164	0.151	–1.086	0.278	
Group 70–79: Frequency	–0.086	0.140	–0.615	0.539	
Group 60–69: Imaginability	–0.040	0.128	–0.312	0.755	
Group 70–79: Imaginability	–0.069	0.118	–0.582	0.561	
Frequency: Imaginability	0.115	0.174	0.663	0.508	
Group 60–69: Frequency: Imaginability	–0.017	0.090	–0.190	0.850	
Group 70–79: Frequency: Imaginability	0.004	0.083	0.046	0.963	

***p < 0.001; **p < 0.01; *p < 0.05.

### Discussion

As in Experiments 1 and 2, the results from Experiment 3 show a substantial effect of aging on word recognition speed. However, unlike the accuracy stabilization observed in the previous experiments, the accuracy level was significantly reduced in the fourth-age group compared to the early aging group. We also found that unlike previous experiments, frequency affected participants’ accuracy. Thus, the cognitive load associated with decision-making after recognition increased the frequency of error in our sample.

## General discussion

The objective of the present study was to establish how the visual recognition of words is affected during the fourth age when tasks involving different cognitive loads are applied. To do so, the RT and accuracy of third- and fourth-age older adults was compared in a naming task, a priming task and a LDT experiment. We found a significant increase in the RT needed to recognize words among those in the fourth-age group in each of the applied experiments. Accuracy, on the other hand, did not show differences between those in the third- and fourth-age groups when the task involved a low cognitive load (Experiment 1) or presented a prime stimulus that modulated recognition (Experiment 2). However, when the task involved a higher processing cost by incorporating a decisional cognitive factor and generated post lexical semantic activation (Experiment 3), the fourth-age group made significantly more mistakes than did the early aging group (60–69 years).

Additionally, the results of Experiments 1, 2, and 3 showed that the modulation of RT associated with the baseline factor of lexical frequency, confirming the stability of it as a robust predictor for word recognition throughout the life cycle, including advanced aging. Interestingly, our data shows in addition to the stability of this effect, that the difference between low and high frequency words (at least in terms of processing time) increases exponentially with aging across experiments. This suggests that people over 80 years of age might experience a stronger change in their cognitive capacities relative to earlier aging (Margrett et al., 2016). Regarding the secondary lexical variables evaluated in different experiments, our results show a similar pattern for PSF and imaginability as the one we observed for lexical frequency, although less accentuated; the difference between low and high PSF as well as the difference between low and high imaginability increases with age. By contrast, the effects of primes (both semantic and ortho-phonological) appeared to be stable across ages; we found no difference in the effect size of such primes between different stages of aging.

The processing speed deficit observed in the fourth-age group was a predictable effect, a product of the physiological changes (neural and sensory) widely described in aging (Miller et al., 2010; Poon et al., 2010; Margrett et al., 2016). At the neural level, the systematic decrease in neural circuits, the lower availability of neurotransmitters and demyelination, are responsible for the reduction in the general cognitive processing speed in old age (Salthouse, 1996, 1999). These changes could affect word recognition for those in the fourth age by delaying the inhibition of lexical competitors and the subsequent selection of the target word. A similar phenomenon occurs with sensory processing. During the fourth age, dysfunction of vision and hearing increases, causing a diffuse, disaggregated, or partial recording of the signal due to lower discrimination and auditory-visual acuity (DeDe and Knilans, 2016; Whitson et al., 2018; Mick et al., 2021). This results in the presence of incomplete lexical inputs that affect recognition. Therefore, the RT differences experienced by those between the third and fourth ages allow us to assume that neural and sensory deficits continue to increase in advanced stages of old age and are responsible—to some extent—for the significant increase in the RT necessary for people to recognize words during the fourth age.

Alternatively (or in addition), the significant RT increase required by people in the fourth age might correspond to the progressive decline in fluid cognitive abilities throughout the life cycle. Multiple studies report that fluid intelligence deficits impact the capacities of abstract and associative thinking, problem solving, task planning, and mental agility of elderly people (Poon et al., 2010; Mitchell et al., 2013; Margrett et al., 2016; Schubert et al., 2020). In this sense, the RT increase in advanced aging is attributed to the fact that fluid intelligence declines abruptly from the age 80 onward (Margrett et al., 2016), usually accompanied by the generalized loss of cognitive functionality (Miller et al., 2010). This phenomenon translates into a substantial decrease in information-processing speed and the ability to efficiently solve a given task (Baltes and Smith, 2003; Miller et al., 2010; Schubert et al., 2020). As a result, the visual recognition of words is affected, specifically the speed necessary to inhibit lexical competitors and the consecutive selection of the representation corresponding to the sensory input. For instance, according to Zacks and Hasher (1997) attentional and executive functioning deficits increase during the fourth age, altering the inhibition of irrelevant linguistic information. In sum, the fluid intelligence deficit experienced by people in the fourth age (probably in addition to physiological deterioration) seems to have a powerful effect on the speed of word recognition, imposing cognitive constraints. Therefore, the results obtained allow us to affirm that, for people in the fourth age, the time required for lexical access and recognizing words will always increase.

Regarding accuracy, the results showed a striking difference compared to the RT: The response accuracy level remained stable for those in the fourth-age group but only when the task involved a very low (Experiments 1) or a small degree of cognitive load (Experiment 2). This behavior was different, however, when the task involved a greater degree of cognitive load (Experiment 3). In healthy cognitive conditions and when the task demands only lexical resources (Experiment 1) or have a post lexical modulation (Experiment 2), a high level of accuracy is observed throughout old age. For example, according to Ratcliff et al. (2011), older fourth-age individuals are likely more conservative and cautious when recognizing words, which is reflected in slower responses compared to those made by third-age individuals. However, at the same time they execute the process with greater confidence, without taking unnecessary risks, obtaining good accuracy and efficient word recognition, similar to that of their third-age counterparts. From another perspective, in line with the self-regulated model of Stine-Morrow et al., 2006a,b, people in the fourth age may reassign their cognitive resources to processing levels that are more difficult, specifically to levels of visual discrimination and the sensory analysis of the input signal. In this way, the transfer of resources to these levels would reduce the speed of recognition while providing a higher level of assertiveness.

A more specific explanation for the high accuracy during the fourth age is that cognitive aging does not consist solely of a decline. As mentioned in the introduction, along with the reduction in fluid intelligence, older people see a maintenance in their cognitive reserves of experience, knowledge, and vocabulary, so-called crystallized intelligence. Different studies confirm that older adults present good conceptual performance, which allows them to recognize words with a high level of accuracy (Ratcliff et al., 2011; DeDe and Knilans, 2016; Wulff et al., 2016). Therefore, this good performance is associated with the maintenance of semantic skills in old age (Stern, 2009; DeDe and Knilans, 2016; Wulff et al., 2016). Crystallized intelligence continues to grow in advanced stages of aging, since older adults have more information and accumulate more experiences, expanding their cognitive reserves (Stern, 2009; Lojo-Seoane et al., 2014; Margrett et al., 2016). In addition, older adults reorganize their semantic networks, configure new links and connections between lexical pieces (Wulff et al., 2016), and consolidate more stable and robust networks composed of frequent and familiar information, all of which facilitates the recognition of sensory input. In summary, many factors indicate that the development of crystallized intelligence allows the optimization of the general cognitive performance necessary to respond to environmental demands (Cooley, 2020), improving recognition accuracy and enhancing linguistic functionality during the fourth age, counteracting—to some extent—the decline in cognitive and energetic resources (Burke and Shafto, 2008) and fluid intelligence (Margrett et al., 2016).

However, the benefits associated with crystallized intelligence during the fourth age can be reduced when the task requires a greater cognitive demand (Experiment 3). Specifically, when the test incorporated a decisional cognitive factor, the RT obtained included not only the recognition of sensory input but also the time necessary to make the decision. During the LDT, the ability to make a decision occurs after signal recognition (Balota and Chumbley, 1984). Therefore, both the RT and the total cognitive load of the task would be increased, expanding the complexity and amount of cognitive resources deployed to respond to the task. In addition, the higher cost involved in making a decision could facilitate access to the meaning of the recognized word, activating semantic information or the categorical effects derived from the target word. Therefore, when the task incorporates a decisional cognitive factor, it consumes a greater amount of resources and generates a higher cognitive effort, thus negatively impacting the accuracy of word recognition for those in the fourth age.

## Conclusion

The results of the present study contribute to a better understanding of visual word recognition in the final stage of the life cycle. The applied experiments showed a significant increase in RT for those in the fourth age, independent of the cognitive load associated with the task. However, at the same time, stabilization and high level of accuracy were observed when the task did not entail a high cognitive demand. Nevertheless, when the test incorporated the making of a decision, the fourth-age group committed significantly more errors than did the early aging group. These results are consistent with evidence that establishes that cognitive aging presents a distinctive functional configuration, with aspects that show evident deterioration while other aspects seem well preserved. In this process, the fluid intelligence deficit significantly affects the RT needed to recognize words. Conversely, the maintenance in crystallized intelligence strengthens the accuracy of the process, which maintain linguistic functionality to a certain extent in advanced stages of old age. We believe that the results obtained should be considered basic empirical evidence in the visual recognition of words during the fourth age.

A limitation of our study is that we explored only visual word recognition leaving the question of whether the observed findings would extrapolate to other modalities, such as auditory word recognition. Indeed, future research should address this question. Another important further development should be the exploration of the (neuro)physiological correlates of the lexical behavior described in this study, expanding the knowledge of the way in which older adults of advanced age manifest their cognitive and linguistic changes. Finally, we believe that it is necessary to expand this research to production studies, specifically word recovery in the oldest among the older adults, considering that production skills are typically affected during aging.

## Data availability statement

The raw data supporting the conclusions of this article will be made available by the authors, without undue reservation.

## Ethics statement

The studies involving human participants were reviewed and approved by University’s Ethics, Bioethics and Biosafety Committee. ID: 21170718. The patients/participants provided their written informed consent to participate in this study.

## Author contributions

CR: conceptualization, methodology, data curation, data analysis, original draft preparation, and writing—reviewing and editing. BR: methodology, data analysis, original draft preparation, and writing—reviewing and editing. EG: software, data curation, data analysis, visualization, original draft preparation, writing—reviewing and editing. All authors contributed to the article and approved the submitted version.

## Footnotes

1
https://www.bcbl.eu/databases/espal/
