# Supplementary material for: Visual word recognition among oldest old people: The effect of age and cognitive load
Source: Front Aging Neurosci. 2022 Sep 30;14:1007048. doi: 10.3389/fnagi.2022.1007048 (PMC9561928; doi:10.3389/fnagi.2022.1007048)
Supplement: Supplementary file 1 [file Data_Sheet_1.docx]

**Appendix A**

Table A1. Experimental items and fillers for Experiment 1

| **Item** | **Visual stimulus** | **Frequency** | **Syllabic frequency** |
| --- | --- | --- | --- |
| 1 | Factiples | No word | No word |
| 2 | Dilatar | Low | High |
| 3 | Panar | No word | No word |
| 4 | Cloana | Filler stimuli | Filler stimuli |
| 5 | Mucosas | Low | High |
| 6 | Rodino | Filler stimuli | Filler stimuli |
| 7 | Distal | Low | High |
| 8 | Calar | Low | High |
| 9 | Palabras | High | High |
| 10 | Cría | High | Low |
| 11 | Signo | High | Low |
| 12 | Razón | Filler stimuli | Filler stimuli |
| 13 | Codorfiz | No word | No word |
| 14 | Múltiplos | Low | Low |
| 15 | Trono | High | Low |
| 16 | Meles | Filler stimuli | Filler stimuli |
| 17 | Montaca | No word | No word |
| 18 | Balmío | No word | No word |
| 19 | Bronce | High | Low |
| 20 | Abenul | Filler stimuli | Filler stimuli |
| 21 | Bruyo | No word | No word |
| 22 | Balsero | Low | Low |
| 23 | Castino | No word | No word |
| 24 | Cabena | No word | No word |
| 25 | Clocar | No word | No word |
| 26 | Sicario | Low | High |
| 27 | Medio | Filler stimuli | Filler stimuli |
| 28 | Animal | High | High |
| 29 | Garfio | Low | Low |
| 30 | Frumas | Filler stimuli | Filler stimuli |
| 31 | Tricu | No word | No word |
| 32 | Barno | No word | No word |
| 33 | Polvorín | Low | Low |
| 34 | Frates | No word | No word |
| 35 | Problema | High | High |
| 36 | Médico | High | High |
| 37 | Cruno | No word | No word |
| 38 | Nótel | No word | No word |
| 39 | Rebatir | Filler stimuli | Filler stimuli |
| 40 | Burdel | Filler stimuli | Filler stimuli |
| 41 | Comer | High | High |
| 42 | Empuñar | Low | High |
| 43 | Milicar | No word | No word |
| 44 | Carroña | Filler stimuli | Filler stimuli |
| 45 | Profetar | No word | No word |
| 46 | Tifus | Low | High |
| 47 | Trecua | No word | No word |
| 48 | Capcan | No word | No word |
| 49 | Bloque | Filler stimuli | Filler stimuli |
| 50 | Tomar | High | High |
| 51 | Bastinor | No word | No word |
| 52 | Minisclo | No word | No word |
| 53 | Colegio | High | High |
| 54 | Croacia | Filler stimuli | Filler stimuli |
| 55 | Bulías | No word | No word |
| 56 | Abierco | No word | No word |
| 57 | Jardines | High | Low |
| 58 | Casa | High | High |
| 59 | Brotado | Low | Low |
| 60 | Cotor | Filler stimuli | Filler stimuli |
| 61 | Lotal | No word | No word |
| 62 | Sater | No word | No word |
| 63 | Yela | No word | No word |
| 64 | Brillanque | Filler stimuli | Filler stimuli |
| 65 | Encías | Low | High |
| 66 | Franela | Filler stimuli | Filler stimuli |
| 67 | Músina | No word | No word |
| 68 | Macato | No word | No word |
| 69 | Palnar | No word | No word |
| 70 | Croquis | Low | Low |
| 71 | Rencillas | Low | Low |
| 72 | Infecnar | No word | No word |
| 73 | Rebanir | No word | No word |
| 74 | Olvidar | High | Low |
| 75 | Bravo | High | Low |
| 76 | Médito | No word | No word |
| 77 | Clonar | Low | Low |
| 78 | Troyo | No word | No word |
| 79 | Bronte | No word | No word |
| 80 | Colemio | No word | No word |
| 81 | Rencidas | No word | No word |
| 82 | Frases | High | Low |
| 83 | Mucotas | No word | No word |
| 84 | Tribu | High | Low |
| 85 | Yerra | Low | Low |
| 86 | Militar | High | High |
| 87 | Infectar | Low | High |
| 88 | Brillante | Filler stimuli | Filler stimuli |
| 89 | Múltinos | No word | No word |
| 90 | Burnel | Filler stimuli | Filler stimuli |
| 91 | Montaña | High | Low |
| 92 | Brotafo | No word | No word |
| 93 | Tillus | No word | No word |
| 94 | Cloaca | Filler stimuli | Filler stimuli |
| 95 | Bría | No word | No word |
| 96 | Frutas | Filler stimuli | Filler stimuli |
| 97 | Bramo | No word | No word |
| 98 | Mefio | Filler stimuli | Filler stimuli |
| 99 | Crudo | High | Low |
| 100 | Abedul | Filler stimuli | Filler stimuli |
| 101 | Coler | No word | No word |
| 102 | Nobel | Low | High |
| 103 | Dilanar | No word | No word |
| 104 | Sigco | No word | No word |
| 105 | Música | High | High |
| 106 | Bruto | High | Low |
| 107 | Color | Filler stimuli | Filler stimuli |
| 108 | Carroca | Filler stimuli | Filler stimuli |
| 109 | Abierto | High | High |
| 110 | Blote | Filler stimuli | Filler stimuli |
| 111 | Bujías | Low | Low |
| 112 | Saber | High | High |
| 113 | Garmio | No word | No word |
| 114 | Canar | No word | No word |
| 115 | Macaco | Low | High |
| 116 | Cabeza | High | High |
| 117 | Codorniz | Low | High |
| 118 | Tregua | High | Low |
| 119 | Racón | Filler stimuli | Filler stimuli |
| 120 | Franena | Filler stimuli | Filler stimuli |
| 121 | Polvomín | No word | No word |
| 122 | Pajar | Low | High |
| 123 | Captan | Low | Low |
| 124 | Local | High | High |
| 125 | Cromis | No word | No word |
| 126 | Rebanir | Filler stimuli | Filler stimuli |
| 127 | Probleja | No word | No word |
| 128 | Olvinar | No word | No word |
| 129 | Enmías | No word | No word |
| 130 | Jardifes | No word | No word |
| 131 | Meses | Filler stimuli | Filler stimuli |
| 132 | Tolar | No word | No word |
| 133 | Profesar | Low | High |
| 134 | Castigo | High | Low |
| 135 | Cafa | No word | No word |
| 136 | Disnal | No word | No word |
| 137 | Barco | High | Low |
| 138 | Bastidor | Low | Low |
| 139 | Anital | No word | No word |
| 140 | Sicanio | No word | No word |
| 141 | Ministro | High | High |
| 142 | Rodillo | Filler stimuli | Filler stimuli |
| 143 | Rebatir | Low | High |
| 144 | Balseno | No word | No word |
| 145 | Palpar | Low | Low |
| 146 | Empugar | No word | No word |
| 147 | Factibles | Low | Low |
| 148 | Palafras | No word | No word |
| 149 | Baldío | Low | Low |
| 150 | Croania | Filler stimuli | Filler stimuli |

**Appendix B**

Table B1. Experimental items and fillers for Experiment 2

| **Item** | | **Prime**  **(visual word)** | **Target**  **(visual word)** | **Frequency** | **Type of prime** | **Trial/Filler** |
| --- | --- | --- | --- | --- | --- | --- |
| 1 | Hospital | | Doctor | High | Semantic | Trial |
| 2 | Grillo | | Zurda | Low | Not related | Trial |
| 3 | Moler | | Templar | Low | Not related | Trial |
| 4 | Tocar | | Topar | Low | Phonological | Trial |
| 5 | Guirnalda | | Acción | High | Not related | Filler stimuli |
| 6 | Cepillo | | Cielo | High | Not related | Trial |
| 7 | Poroto | | Frijol | Low | Semantic | Trial |
| 8 | Ayuda | | Favor | High | Semantic | Filler stimuli |
| 9 | Correr | | Comer | High | Phonological | Trial |
| 10 | Flotan | | Culpa | High | Not related | Trial |
| 11 | Tenor | | Terror | High | Phonological | Trial |
| 12 | Sidra | | Día | High | Not related | Trial |
| 13 | Colmena | | Panal | Low | Semantic | Trial |
| 14 | Águila | | Padre | High | Not related | Filler stimuli |
| 15 | Océano | | Isla | High | Semantic | Filler stimuli |
| 16 | Aguijón | | Venta | High | Not related | Trial |
| 17 | Abril | | Atril | Low | Phonological | Trial |
| 18 | Gritón | | Puercos | Low | Not related | Trial |
| 19 | Beso | | Parco | Low | Not related | Trial |
| 20 | Flores | | Planta | High | Semantic | Trial |
| 21 | Campo | | Rural | High | Semantic | Trial |
| 22 | Cordel | | Púgil | Low | Not related | Filler stimuli |
| 23 | Calzas | | Asar | Low | Not related | Trial |
| 24 | Conocer | | Saber | High | Semantic | Filler stimuli |
| 25 | Hebra | | Luna | High | Not related | Trial |
| 26 | Maní | | Japón | High | Not related | Trial |
| 27 | Cuenca | | Cuenta | High | Phonological | Trial |
| 28 | Filtro | | Jarrón | Low | Not related | Trial |
| 29 | Blanco | | Albo | Low | Semantic | Trial |
| 30 | Surgir | | Surtir | Low | Phonological | Trial |
| 31 | Furgón | | Liga | High | Not related | Trial |
| 32 | Mellizo | | Copa | High | Not related | Trial |
| 33 | Lobo | | Calar | Low | Not related | Trial |
| 34 | Lupa | | Poda | Low | Not related | Filler stimuli |
| 35 | Añeja | | Rancia | Low | Semantic | Filler stimuli |
| 36 | Caballero | | Señor | High | Semantic | Trial |
| 37 | Tocar | | Palpar | Low | Semantic | Trial |
| 38 | Hurtar | | Hurgar | Low | Phonological | Trial |
| 39 | Junco | | Junto | High | Phonological | Trial |
| 40 | Tiesto | | Helar | Low | Not related | Trial |
| 41 | Sudar | | Bajón | Low | Not related | Trial |
| 42 | Chillar | | Aullar | Low | Semantic | Filler stimuli |
| 43 | Diurno | | Banda | High | Not related | Trial |
| 44 | Alambrar | | Cercar | Low | Semantic | Trial |
| 45 | Tostada | | Cojín | Low | Not related | Trial |
| 46 | Alcanzó | | Frotó | Low | Not related | Filler stimuli |
| 47 | Pequé | | Pegué | Low | Phonological | Trial |
| 48 | Cuanto | | Cuando | High | Phonological | Trial |
| 49 | Látex | | Verdad | High | Not related | Trial |
| 50 | Población | | Barrio | High | Semantic | Trial |
| 51 | Trombo | | Trompo | Low | Phonological | Trial |
| 52 | Combo | | Suelo | High | Not related | Filler stimuli |
| 53 | Picota | | Diario | High | Not related | Trial |
| 54 | Hierro | | Hielo | High | Phonological | Trial |
| 55 | Tarot | | Llevar | High | Not related | Trial |
| 56 | Tambo | | Cirio | Low | Not related | Trial |
| 57 | Sustraer | | Hurtar | Low | Semantic | Trial |
| 58 | Pulpo | | Rajó | Low | Not related | Trial |
| 59 | Combate | | Guerra | High | Semantic | Trial |
| 60 | Grumo | | Grupo | High | Phonological | Filler stimuli |
| 61 | Príncipe | | Reyes | High | Semantic | Trial |
| 62 | Renca | | Renta | High | Phonological | Trial |
| 63 | Duchas | | Blanco | High | Not related | Trial |
| 64 | Teme | | Hierve | Low | Not related | Trial |
| 65 | Demoler | | Cuento | High | Not related | Trial |
| 66 | Disolver | | Diluir | Low | Semantic | Trial |
| 67 | Tacón | | Libre | High | Not related | Filler stimuli |
| 68 | Brindar | | Brincar | Low | Phonological | Trial |
| 69 | Pito | | Tienta | Low | Not related | Trial |
| 70 | Cumple | | Cumbre | High | Phonological | Filler stimuli |
| 71 | Muela | | Lanas | Low | Not related | Trial |
| 72 | Aro | | Grifos | Low | Not related | Filler stimuli |
| 73 | Zarza | | Zarpa | Low | Phonological | Trial |
| 74 | Tormenta | | Viento | High | Semantic | Trial |
| 75 | Mantel | | Lucha | High | Not related | Trial |
| 76 | Castor | | Marca | High | Not related | Trial |
| 77 | Cuerno | | Cuerpo | High | Phonological | Trial |
| 78 | Huerta | | Vergel | Low | Semantic | Trial |
| 79 | Braga | | Betún | Low | Not related | Trial |
| 80 | Rubia | | Rusia | High | Phonological | Filler stimuli |
| 81 | Saco | | Costal | Low | Semantic | Trial |
| 82 | Bandas | | Bancas | Low | Phonological | Trial |
| 83 | Suela | | Morral | Low | Not related | Trial |
| 84 | Tratan | | Trazan | Low | Phonological | Filler stimuli |
| 85 | Lealtad | | Amor | High | Semantic | Trial |
| 86 | Ciprés | | Carne | High | Not related | Trial |
| 87 | Vello | | Docta | Low | Not related | Filler stimuli |
| 88 | Clavel | | Sala | High | Not related | Trial |
| 89 | Cotizar | | Pinchar | Low | Not related | Trial |
| 90 | Falsa | | Falta | High | Phonological | Trial |
| 91 | Luto | | Fuman | Low | Not related | Trial |
| 92 | Virgo | | Poco | High | Not related | Trial |
| 93 | Asma | | Aspa | Low | Phonological | Filler stimuli |
| 94 | Nota | | Pillar | Low | Not related | Filler stimuli |
| 95 | Carraspear | | Toser | Low | Semantic | Trial |
| 96 | Gorila | | Reúnen | Low | Not related | Trial |
| 97 | Junio | | Abril | High | Semantic | Trial |
| 98 | Gala | | Gana | High | Phonological | Trial |
| 99 | Animal | | Arte | High | Not related | Trial |
| 100 | Mandar | | Manchar | Low | Phonological | Trial |

**Appendix C**

Table C1. Experimental items and fillers for Experiment 3

| **Item** | **Visual stimulus** | **Frequency** | **Imaginability** |
| --- | --- | --- | --- |
| 1 | Bizcocho | Low | High |
| 2 | Usurpar | Filler stimuli | Filler stimuli |
| 3 | Biomfo | No word | No word |
| 4 | Conciso | Low | Low |
| 5 | Cieno | No word | No word |
| 6 | Carraz | No word | No word |
| 7 | Causa | High | Low |
| 8 | Madeya | No word | No word |
| 9 | Zornal | No word | No word |
| 10 | Libro | High | High |
| 11 | Morral | Low | High |
| 12 | Esternón | Filler stimuli | Filler stimuli |
| 13 | Voluncad | No word | No word |
| 14 | Valoyes | No word | No word |
| 15 | Hina | No word | No word |
| 16 | Sadisto | No word | No word |
| 17 | Pompono | Filler stimuli | Filler stimuli |
| 18 | Dormir | Filler stimuli | Filler stimuli |
| 19 | Concientia | No word | No word |
| 20 | Carácter | High | Low |
| 21 | Equino | Low | High |
| 22 | Senfación | No word | No word |
| 23 | Luna | High | High |
| 24 | Comifa | No word | No word |
| 25 | Certena | No word | No word |
| 26 | Sinio | No word | No word |
| 27 | Percepción | High | Low |
| 28 | Monal | No word | No word |
| 29 | Fronar | Filler stimuli | Filler stimuli |
| 30 | Niña | High | High |
| 31 | Brasa | Low | High |
| 32 | Agua | High | High |
| 33 | Joroba | Low | High |
| 34 | Corazón | High | High |
| 35 | Comultar | Filler stimuli | Filler stimuli |
| 36 | Indaga | Low | Low |
| 37 | Veraco | No word | No word |
| 38 | Pérfido | Low | Low |
| 39 | Lenfua | Filler stimuli | Filler stimuli |
| 40 | Parne | No word | No word |
| 41 | Alarne | No word | No word |
| 42 | Médino | No word | No word |
| 43 | Almeja | Low | High |
| 44 | Deteo | Filler stimuli | Filler stimuli |
| 45 | Farsante | Filler stimuli | Filler stimuli |
| 46 | Agonio | No word | No word |
| 47 | Humor | Filler stimuli | Filler stimuli |
| 48 | Madela | No word | No word |
| 49 | Belún | No word | No word |
| 50 | Exisne | Filler stimuli | Filler stimuli |
| 51 | Bistuní | No word | No word |
| 52 | Lógiba | No word | No word |
| 53 | Fuego | Filler stimuli | Filler stimuli |
| 54 | Abruma | Low | Low |
| 55 | Especial | Filler stimuli | Filler stimuli |
| 56 | Burdo | Low | Low |
| 57 | Ampolla | Filler stimuli | Filler stimuli |
| 58 | Cierto | High | Low |
| 59 | Tortuoso | Low | Low |
| 60 | Ranón | No word | No word |
| 61 | Cuatioya | No word | No word |
| 62 | Intención | High | Low |
| 63 | Diaclo | Filler stimuli | Filler stimuli |
| 64 | Macena | No word | No word |
| 65 | Fascita | No word | No word |
| 66 | Morboso | Low | Low |
| 67 | Real | High | Low |
| 68 | Recurso | High | Low |
| 69 | Melona | No word | No word |
| 70 | Manos | High | High |
| 71 | Sórdifo | No word | No word |
| 72 | Cuerpo | High | High |
| 73 | Mujer | High | High |
| 74 | Molusco | Low | High |
| 75 | Cubeta | Low | High |
| 76 | Médico | High | High |
| 77 | Mabos | No word | No word |
| 78 | Abruya | No word | No word |
| 79 | Sórdido | Low | Low |
| 80 | Caráccer | No word | No word |
| 81 | Betún | Low | High |
| 82 | Usurlar | Filler stimuli | Filler stimuli |
| 83 | Pérfiyo | No word | No word |
| 84 | Madera | High | High |
| 85 | Cubena | No word | No word |
| 86 | Voluntad | High | Low |
| 87 | Maceta | Low | High |
| 88 | Recurno | No word | No word |
| 89 | Bizcoto | No word | No word |
| 90 | Esterfón | Filler stimuli | Filler stimuli |
| 91 | Molusno | No word | No word |
| 92 | Pomposo | Filler stimuli | Filler stimuli |
| 93 | Motal | No word | No word |
| 94 | Muler | No word | No word |
| 95 | Farsanbe | Filler stimuli | Filler stimuli |
| 96 | Valores | High | Low |
| 97 | Madeja | Low | High |
| 98 | Cuerlo | No word | No word |
| 99 | Burco | No word | No word |
| 100 | Melosa | Low | Low |
| 101 | Fascina | Low | Low |
| 102 | Lógica | High | Low |
| 103 | Brata | No word | No word |
| 104 | Cierno | No word | No word |
| 105 | Indana | No word | No word |
| 106 | Licro | No word | No word |
| 107 | Tortuono | No word | No word |
| 108 | Hija | High | High |
| 109 | Sensación | High | Low |
| 110 | Lengua | Filler stimuli | Filler stimuli |
| 111 | Luva | No word | No word |
| 112 | Comida | High | High |
| 113 | Niya | No word | No word |
| 114 | Biombo | Low | High |
| 115 | Alarde | Low | Low |
| 116 | Hutor | Filler stimuli | Filler stimuli |
| 117 | Espefial | Filler stimuli | Filler stimuli |
| 118 | Cauna | No word | No word |
| 119 | Verano | High | High |
| 120 | Agobio | Low | Low |
| 121 | Morbono | No word | No word |
| 122 | Coralón | No word | No word |
| 123 | Bisturí | Low | High |
| 124 | Capaz | High | Low |
| 125 | Intenfión | No word | No word |
| 126 | Certera | Low | Low |
| 127 | Sadismo | Low | Low |
| 128 | Pernepción | No word | No word |
| 129 | Concino | No word | No word |
| 130 | Moral | High | Low |
| 131 | Ampota | Filler stimuli | Filler stimuli |
| 132 | Atua | No word | No word |
| 133 | Cuantiosa | Low | Low |
| 134 | Dorbir | Filler stimuli | Filler stimuli |
| 135 | Parque | High | High |
| 136 | Deseo | Filler stimuli | Filler stimuli |
| 137 | Equimo | No word | No word |
| 138 | Comulgar | Filler stimuli | Filler stimuli |
| 139 | Zorzal | Low | High |
| 140 | Simio | Low | High |
| 141 | Fueno | Filler stimuli | Filler stimuli |
| 142 | Razón | High | Low |
| 143 | Existe | Filler stimuli | Filler stimuli |
| 144 | Diablo | Filler stimuli | Filler stimuli |
| 145 | Frotar | Filler stimuli | Filler stimuli |
| 146 | Jorota | No word | No word |
| 147 | Conciencia | High | Low |
| 148 | Almena | No word | No word |
| 149 | Cielo | High | High |
| 150 | Rean | No word | No word |
